# Incidence Proportions From Random-Effects Meta-Analyses of Rare Events With Large Cluster Heterogeneity, Including Sensitivity to the Normal Distribution Assumption

**DOI:** 10.7759/cureus.93214

**Published:** 2025-09-25

**Authors:** Franklin Dexter, Rakesh Sondekoppam, Emine O Bayman

**Affiliations:** 1 Department of Anesthesia, University of Iowa, Iowa City, USA; 2 Anesthesiology, Stanford University, Palo Alto, USA; 3 Biostatistics, University of Iowa, Iowa City, USA

**Keywords:** healthcare-associated infection, hospital engineering, industrial engineering, low incidence, meta-analysis, performance appraisal, quality control charts, rare adverse event, statistical model, ultrasound-guided regional anesthesia

## Abstract

Our narrative review examines statistical methods for estimating incidence proportions of rare events (≤5%) with large heterogeneity among clusters. One example dataset is a meta-analysis of incidences of neurological complications after regional anesthesia procedures. The clusters (i.e., studies) are nested within groups, whether they use ultrasound guidance or not. The other example dataset consists of ratings of faculty anesthesiologists (i.e., the clusters represent the raters). We review weighted mean proportions among clusters (i.e., marginal estimates). We also review incidence proportions for the median cluster (i.e., conditional estimates). Because marginal estimates are larger than conditional estimates, we recommend knowing which one is being reported and, for each application, consider which would be preferred.

For both example datasets, the logits of the observed incidences for each cluster followed normal distributions within groups, satisfying statistical assumptions of random-effects logistic regression. Accurate confidence interval coverage for conditional and marginal estimates for incidence proportions can therefore be obtained using random-effects logistic regression. We recommend this method and provide Stata code. Probit regression depends on probits following a normal distribution, which was used for one of the two example datasets.

Calculating incidence proportions for each study individually, along with variance estimates, and then pooling, yields different results (e.g., a 100% relative error in the estimated incidence). We review that the two-step methods for incidence proportions with the closest to nominal confidence interval coverages are those performed with arcsine transformation or the Freeman-Tukey double arcsine, with the inverse calculated based on the harmonic mean sample size. Although these methods are suitable for forest plots, we recommend against using them for primary inference due to their inferior performance compared to random-effects logistic regression.

Hospital management reports using anesthesia data can employ methods that misuse data, neglecting the heterogeneity among clusters (e.g., generalized estimating equations). For example, consider hospital reports of postoperative infections among patients undergoing surgery, which are pooled by surgeons or surgical procedures. Even when there is an absence of interest in the variability of incidences within clusters (e.g., among obstetricians) or even consideration of the clusters (e.g., individual procedures), fixed effects methods (e.g., simply pooling counts) have 95% confidence intervals with coverage <50%, their narrowness falsely suggesting precision. While such situations are generally obvious for meta-analyses of journal articles, often they are not so for managerial applications relevant to anesthesia. We recommend using random-effects models for these administrative and quality improvement reports on rare anesthesia events and large or unmeasured heterogeneity among clusters.

## Introduction and background

Uncommon events in anesthesia, such as infectious complications from regional nerve blocks or accidental dural puncture during labor epidural placement [1], pose challenges for clinical research because of their thankfully low incidence rates (≤5%). Large sample sizes are required to estimate the true incidence of infrequent events with precision (i.e., narrow confidence intervals), often achieved by pooling data from heterogeneous data sources (e.g., different studies in a meta-analysis) [1,2].

A recent random-effects meta-analysis evaluated the incidence proportions of neurological dysfunction lasting more than 48 hours after regional anesthetic nerve block [2]. The estimated overall incidence proportions in the article were 1.2% (95% confidence interval, 0.9-1.5%) with ultrasound guidance and 0.8% (95% confidence interval, 0.6-1.0%) without ultrasound guidance. There were many studies (i.e., clusters) in each of the two groups: 74 in the ultrasound guidance group and 61 in the no-ultrasound group. There were also large heterogeneities in incidence proportions among studies (i.e., “clusters”), with listed I² statistics of 89% for the ultrasound guidance group and 81% for the studies without ultrasound guidance. There were small sample sizes of patients per cluster compared to the observed incidences, with harmonic means of 26 and 21 patients, respectively. Thus, many studies reported zero events of neurological dysfunction. We review the expected biases in the incidence proportions of groups and the coverages of the confidence intervals for groups (i.e., whether calculated 95% confidence intervals include the true incidence for 95% of such meta-analyses). Specifically, we review that confidence intervals, for incidence proportions of groups, calculated using random-effects logistic regression and probit regression, have small bias and accurate coverage. In contrast, such confidence intervals calculated using two-step methods, as shown in forest plots, can be inaccurate. We review two interpretations of incidence proportions. One interpretation is conditional, which refers functionally to the median probability among clusters. The second interpretation is marginal, referring to the weighted mean probability across all clusters.

We also use another earlier dataset, evaluations of anesthesiologists’ quality of clinical supervision (e.g., during cesarean births) [3]. In the first dataset, the clusters are studies [2]. In the second dataset, the clusters are raters [3], specifically anesthesiology residents. In the first dataset, the two groups are defined by whether ultrasound guidance is used or not [2]. In the second dataset, the groups are non-successive years [3]. Just as episodes of neurological dysfunction are uncommon [2], so is the poor quality of clinical supervision while trainees care for anesthetized patients [3]. We utilize the second dataset to expand our recommendations to investigators and journal editors on estimating incidence proportions for rare events in anesthesia data. We review that confidence interval coverage can be inaccurate when heterogeneity is large, and the random effect for variation among clusters does not follow a normal distribution. Such relationships hold not only for meta-analyses (i.e., clusters are comprised of studies) and multi-center clinical trials (e.g., clusters are comprised of enrolling centers), but also for routine hospital reports (e.g., clusters are comprised of rating anesthesiology residents or patients visiting different clinics). The statistical methods are comparable because, for incidence proportions, the counts for each cluster within each group provide complete data, and each cluster falls within only one group. We review the advantages of using random-effects models for these administrative and quality improvement reports.

## Review

Methods

We present specific goals and literature search protocols, following the Scale for the Assessment of Narrative Reviews (SANRA) [4]. Our article is organized and has content to have the maximum score for each of SANRA’s six items. The two example datasets are each fully available in the supplemental PDF file [2,3]. For the dataset of regional anesthesia procedures, the clusters are studies, and the two groups are with and without ultrasound guidance [2]. For the evaluation dataset of binary scores, the clusters consist of anesthesia residents evaluating faculty anesthesiologists, and the two groups are from earlier and later years [3]. As shown in the supplemental PDF file and its accompanying data, each observation (i.e., patient or evaluation) is contained within one cluster (i.e., study or rater), and each cluster belongs exclusively to one group (i.e., ultrasound or no ultrasound, or the earlier or later year). The two studied years are more than four years apart, so raters do not overlap between groups. As numerical results are presented in the article from one successive section to the next, we recommend following along in sequence using the statistical output supplemental PDF (https://doi.org/10.17605/OSF.IO/8FNW6). Our narrative review has two objectives.

First, we review earlier articles that evaluated the extent to which the probability distribution of the random effect matters for unbiased results and correct (i.e., nominal) confidence interval coverage when estimating conditional (median) or marginal (mean) incidence proportions. Nominal 95% confidence intervals are expected to include true values for 95% of replications. We limit our review to population incidence proportions ≤5% (the mathematics is the same for incidence proportions ≥95%).

Second, multiple methods for calculating marginal predictive probabilities do not model the heterogeneity among studies (e.g., generalized estimating equations). When presented for meta-analyses, investigators and journal editors would promptly recognize that the assumptions of such fixed effects analyses are invalid [2]. However, these methods are routinely applied for retrospective cohort, managerial epidemiology, and internal hospital engineering projects without considering the assumptions for variations among clusters. Statistical methods for robust variance estimation (e.g., Huber-White sandwich estimators, as used in generalized estimating equations) are robust to misspecification of the correlations among patients within clusters, but not to errors in the model of associations among clusters. We review that when quantifying incidences of rare events, even for routine hospital reports, generally random-effects methods analogous to meta-analyses should be used. Our datasets show that simply pooling the data (i.e., fixed effects models) provides biased (i.e., invalid) marginal estimates of incidence proportions under conditions of uncommon anesthesia events.

The Supplemental Excel file (https://doi.org/10.17605/OSF.IO/8FNW6) details the results of the three Scopus searches performed in March 2025 to prepare our narrative review. For example, the first search was TITLE(“meta-analysis” OR “meta-regression”) AND TITLE(“incidence” OR “prevalence” OR “single proportion”) AND TITLE-ABS(“Monte-Carlo” OR simulat*). This first search is found in the worksheet labeled “Scopus 1.” Our goal in these worksheets is to show that we aimed to include every article, of any statistical method, that utilized computer simulations to demonstrate accurate confidence (or credible) interval coverage for marginal or conditional estimates of pooled uncommon proportions with large heterogeneity among clusters. 

Logits and probits following a normal distribution among clusters

**Figure 1:**
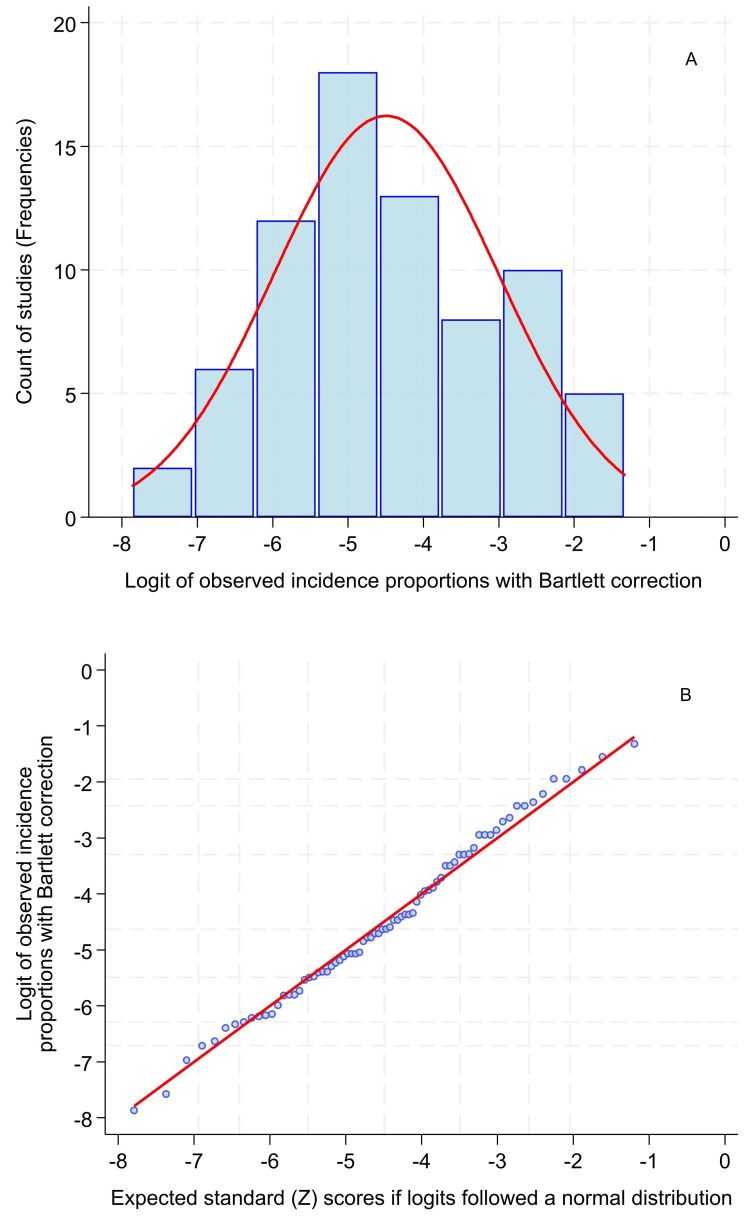
Logits followed a normal distribution In the normal quantile plot, grid lines are at the 5^th^, 10^th^, 25^th^, 50^th^, 75^th^, 90^th^, and 95^th^ percentiles. In the histogram (panel A), grid lines are at the major tick marks. The sample size equals N = 74 clusters, which is the larger of the two sample sizes from the study of regional anesthesia procedure complications [2]. The straight line in the normal quantile plot (panel B) indicates that the logits followed a normal distribution. The Shapiro-Wilk W statistic equals 0.99, showing that it is close to a normal distribution (P = 0.66).

A supplemental file, section Stata A, lists the numerators and denominators for all the clusters in the regional anesthesia groups, obtained from Reference [2]’s third figure (with ultrasound guidance) and Reference [2]’s fourth figure (without ultrasound guidance) [2]. Each cluster (i.e., study) is reproduced in the supplemental Excel worksheet labeled “Lemke.” Figure 1 displays a histogram and probability plot for the logits of the proportions of patients with neurological complications lasting more than 48 hours among the procedures performed with ultrasound (Stata B) [2,5]. The logit equals the natural logarithm of a ratio, where the numerator is the observed proportion (p) and the denominator is one minus the observed proportion. Bartlett’s correction was applied for the multiple clusters with numerators equal to zero [5]. See the Stata supplemental PDF for details. Briefly, clusters with numerators equal to zero had their numerators replaced with one event and their denominators replaced with four times their observed counts.

The resulting logits followed normal distributions (e.g., without ultrasound, Shapiro-Wilk W = 0.98, P = 0.67). These results are unlikely to be type II errors because both groups had more than the N = 50 clusters, which is sufficient for the Shapiro-Wilk test to have approximately 90% statistical power to detect deviation from a normal distribution based on a P-value less than 0.10 [6].

Following our recognition that logits followed a normal distribution, we applied a random-effect logistic regression model. In Stata C, we label each cluster “StudyID” and provide its “Numerator” and “Denominator:”

melogit Numerator || StudyID:, binomial(Denominator)

Stata’s output is in the logit scale. To obtain the incidence proportion, we take the inverse of the logit. The calculation takes two lines of code. The estimated incidence proportions for the median clusters of each group were 0.61% (95% confidence interval, 0.34-1.07%) with ultrasound and 0.64% (0.36-1.16%) without ultrasound. The two groups’ clusters can be compared (Stata D):

melogit Numerator Ultrasound || StudyID:, binomial(Denominator)

Results from that command show no significant difference between studies with ultrasound guidance and those without (odds ratio 0.90, 95% confidence interval 0.42-1.90, P = 0.78) in neurological complications within 48 hours of the regional anesthetic procedures [2].

We hope that we have successfully shown straightforward methods and clear results. They do not match the earlier article, despite using the same data (Stata A) [2]. The earlier article’s calculated incidence proportions among clusters with ultrasound were not 0.61% (95% confidence interval, 0.34-1.07%) but 1.2% (0.9-1.5%) [2]. The earlier results without ultrasound were not 0.64% (0.36-1.16%) but 0.8% (0.6-1.0%). The earlier [2] estimate for the ultrasound group was double that calculated using random-effects logistic regression, where 2.0 = 1.2%/0.61%. This difference was not an issue of reproducibility because the methods were explained [2]. Rather, the issue is the statistical methods employed, which motivates the current article. The difference matters because, in the earlier article [2], the estimate for the ultrasound group of 1.2% does not even fall within the confidence interval for the other, non-ultrasound, group: 0.6-1.0%. Similarly, the estimate for non-ultrasound of 0.8% does not lie within the interval for ultrasound: 0.9-1.5%. The implication is that when performing meta-analyses for rare anesthetic complications, the methods of analysis dramatically affect estimates.

Marginal and conditional estimates

One possible explanation for the different results is that the random-effects logistic regression reported conditional estimates, not marginal estimates. Specifically, a conditional estimate refers to the incidence proportion conditional on the random effect term equaling zero (i.e., reports the incidence proportion for the median cluster, meaning study, and its confidence interval). In contrast, the marginal estimate refers to the weighted mean proportion among clusters [7]. Because the incidence proportions cannot be less than zero, and many observed proportions equaled zero [2], the mean incidence (marginal estimate) exceeded the median incidence (conditional estimate). If the question is the effect of the group on the population, that would be the marginal estimate. If the question is about the effect of the group on the median (or average) cluster, that would be the conditional estimate. For the regional anesthetic studies, marginal estimates were the goal [2]. For estimating overall incidences of complications, generally marginal estimates would be the goal. For the assessment of anesthesia residents’ evaluations, patients’ evaluations, and so on, generally, conditional effects are desired, showing the perceptions of the “average” resident or patient [3].

Due to the mathematical properties of the probit transformation, a single command can estimate the mean after random-effects probit regression [8]. Probit regression was suitable because probit transformations of the observed incidence proportions, with Bartlett’s correction [5], followed normal distributions (Stata E). The marginal estimates were 2.02% (95% confidence interval, 1.04-3.00%) with ultrasound and 2.27% (1.09-3.45%) without ultrasound. These calculations do not match the reported results from the original article, again 1.2% (0.9-1.5%) and 0.8% (0.6-1.0%), respectively. With the assumptions of probit regression satisfied, the marginal (i.e., mean) estimate for short-term neurological complications when procedures were performed without ultrasound was 2.27%, 2.8 times larger than the earlier reported value of 0.8% [2]. This was not due to an error in the earlier article [2], but rather to the calculations performed.

Marginal estimates can also be calculated with random-effects logistic regression (Stata F) [7]. The resulting estimates using logistic regression were 2.17% (95% confidence interval, 1.06-3.27%) with ultrasound and 2.38% (1.11-3.65%) without ultrasound. These results were similar to those from the probit regression in the preceding paragraph, but were not identical. These results also differed considerably from those in the earlier article [2]. As expected, the estimates and confidence intervals differ because the models have different assumptions. One model assumes that the logits are normally distributed, and the other model assumes that the probits are normally distributed.

As we proceed, we emphasize again that all computer code is in sequence in the Stata file. Summaries of the results are not addressable in brief tables because they are a series of calculations. Please follow along in the supplemental: https://doi.org/10.17605/OSF.IO/8FNW6.

Two-step methods are not suitable alternatives to avoid the distributional assumption

Lin and Chu compared the performance of random-effects logistic and probit regression for conditional estimates of incidence proportions [9]. They performed simulations for different heterogeneity estimates (I²), which conceptually represent the percentages of the total variation in the proportions among clusters that were due to true differences among the clusters, as compared with sampling error. When the proportions’ incidences were 1.0% (i.e., similar to the regional anesthesia data [2]) and there were 30 clusters, both the logit and probit methods were unbiased, and their confidence intervals had similar coverage: 96% for I² statistic of 3% and 93% for I² of 43% [9]. (The standard errors were 0.7% for all their coverage estimates, so neither 96% nor 93% probably differed from the nominal 95%.) The implication is that we can trust confidence intervals for both random-effects logistic regression and probit regression for the regional anesthesia procedures dataset [2].

Notice that we have not included whether Lin and Chu [9] studied meta-analyses (i.e., clusters are studies), routine hospital management reports (e.g., clusters are raters), or multi-center clinical trials (e.g., clusters are enrolling centers). This does not matter mathematically because, for incidence proportions, the counts for each cluster within each group provide complete data, and each cluster falls within only one group.

Lin and Chu’s simulations are especially helpful because they included other methods of estimating incidence proportions and confidence intervals [9]. Specifically, there are the so-called two-step methods, as displayed in most meta-analysis forest plots. They first transformed the proportions from each cluster and then used maximum likelihood estimation to pool the transformed estimates across clusters [9]. An inverse transform is used for reporting in the desired probability scale [9]. Transformations are used because otherwise clusters with the smallest observed proportions will generally have smaller variances in the proportion scale, resulting in those clusters receiving disproportionate weight from the pooling based on the inverse of the variances [10]. Doing so results in underestimating the true incidence proportions and poor confidence interval coverage [10]. For incidence proportions of 1.0%, the two categories of transforms with the best performance based on bias and confidence interval coverage were the arcsine transformation and the Freeman-Tukey double arcsine, with the inverse calculated based on the harmonic mean sample size [9,11]. Increasing the variability among clusters (I²) from 3% to 43% resulted in larger bias and less accurate confidence interval coverage for both [9]. Decreasing the harmonic mean sample size of patients per cluster from 2190 to 219 also resulted in a larger bias and less accurate coverage [9]. For the combination of I² = 43% and a harmonic mean of 219 patients (coefficient of quartile deviation of the sample size of 33%), logistic and probit mixed models were unbiased and had 93% confidence interval coverage [9]. In contrast, the arcsine and double arcsine transformations yielded biased estimates, overestimating the true proportions by 0.17 and 0.13, respectively [9]. They had poor confidence interval coverage, 81% and 87%, differing significantly from the nominal 95% coverage [9]. Even with the absence of heterogeneity among clusters and an imbalance in sample sizes among clusters, decreasing the incidence proportions from 1.0% to 0.5% or even 0.1% resulted in the arcsine transformation’s confidence interval coverage being substantially (< 10%) less than nominal [5]. The arcsine had, however, better performance at low incidences than using no transformation [12].

For context, the regional anesthesia data had coefficients of quartile deviation of sample sizes larger than the 33% simulated by Lin and Chu’s [9]: 62% with ultrasound guidance and 85% without ultrasound (Stata G) [2]. The sample sizes of the clusters were smaller than the harmonic mean of 219 patients simulated by Lin and Chu [9]: 26 for patients with ultrasound and 21 without ultrasound [2]. Finally, the heterogeneity I² statistics from the double arcsine-transformed data were larger than the 43% simulated by Lin and Chu [9]: I² = 92% for clusters in the ultrasound group and I² = 95% without ultrasound [2].

We used the regional anesthesia dataset [2], performed Freeman-Tukey transformations, and then maximum likelihood estimation for the random-effects meta-analysis. We subsequently applied the harmonic mean to calculate the inverse, matching the preceding result from Lin and Chu (Stata H). The estimates for incidence proportions were implausibly low, at 0.3% (95% confidence interval, 0.0-0.9%) for clusters with ultrasound and 0.1% (0.0-0.7%) for clusters without ultrasound. These should be marginal estimates, not conditional, but they are several-fold too small for both. The best-performing method from Lin and Chu [9] failed in the regional anesthesia application. We recommend this be a major lesson. Regardless of whether performing meta-analyses or simply averaging accidental dural puncture rates among trainees of various levels of experience, do not use the two-stage methods except for graphical purposes.

The reason why the estimates are too small is understood from Hamza et al., who examined two-stage methods under conditions relevant to our anesthesia studies [2,3], with small incidence proportions and large heterogeneity characteristics [13]. Specifically, their simulations used 50 clusters, with a within-cluster sample size mean of 40 and a coefficient of quartile deviation among clusters of 51% [13]. The true median incidence proportion equaled 7% [13]. The I² was approximately 91% [13]. Thus, the numbers of clusters, sample sizes within clusters, and heterogeneity among clusters matched the anesthesia data [2]. However, the incidence proportions were larger (i.e., the simulations will obtain better confidence interval coverage) [13]. Nevertheless, using the logit transformation, followed by restricted maximum likelihood estimation, asymptotic Wald confidence intervals, and then inverse transformation, the incidence proportion confidence intervals had coverage of only 10% (standard error 1%). In contrast, for the same conditions, random-effects logistic regression with the same asymptotic confidence interval achieved nominal (94%) coverage (standard error of 1%) [13]. The extremely poor coverage with the two-step methods occurred in the settings of uncommon binary events and large heterogeneity among clusters [9,13]. This explains the difference between our results and those of the earlier article [2].

Generalized estimating equations are insufficient to address the distributional assumption issue

We have so far presented two approaches for obtaining the marginal (mean) estimates of the incidence proportion using regional procedure meta-analysis data, random-effects probit regression, and random-effects logistic regression, followed by running the margins command (Stata). For these anesthesia meta-analysis data [2], with I² values of 92% (ultrasound) and 95% (without), it would be obvious to investigators and journal statistical editors that random effects rather than fixed effects would be more suitable for the meta-analysis.

For the current section, we continue to use the regional anesthesia data [2], but we envision different applications with similar incidence proportions, sample sizes, and heterogeneity statistics. Would the need for random-effects analysis be clear? For example, a hospital analyst is asked to calculate the current incidence of postoperative infections among patients undergoing surgery preceded by a regional anesthesia procedure [14,15]. The desired estimate should equal the ratio of the total infections to total cases (i.e., marginal mean), but with a standard error or a confidence interval. Using the Freeman-Tukey double arcsine transformation and DerSimonian and Laird random effect, the I² is approximately 98%. While the need for a random-effects analysis would be apparent for meta-analysis [14,15], we ask readers: has anyone seen a random-effects meta-analysis technique applied at their hospitals for such data? A major goal of our review is to emphasize that, when estimating rare events, one should use random-effects methods unless it is known that they are unnecessary. Evidence that random-effects methods are unnecessary is not the presence of many clusters with zero numerators (i.e., what the preceding and following results show is the lack of validity of assuming homogeneity among clusters if many have insufficient sample sizes relative to the incidence proportions). If the sample sizes are small (i.e., many zero numerators), our work highlights the need to rely on earlier scientific studies to judge the I² or, alternatively, assume that it is large.

One approach to estimate a standard error for the pooled (marginal) incidence proportion would be to use survey methods, with each operating room, surgeon, anesthesiologist, or rater treated as a cluster. The jackknife estimator iteratively removes one cluster at a time, using variation in the replicated ratios to estimate the variance. Removing one cluster at a time [2], the jackknife estimated mean incidence proportion equaled 1.67% (95% confidence interval, −1.62% to 4.96%) for procedures performed with ultrasound guidance and 0.92% (−0.02% to 1.87%) without (Stata I). The lower limits of the confidence intervals for the groups (−1.62% and −0.02%) were negative because the incidence proportions were small (i.e., the topic of the current article), and this statistical method has not assumed a binomial distribution for within-cluster variation. The confidence intervals are inaccurate because incidences cannot be negative values. However, the same calculations performed using the published infection data among operating rooms give 3.8% (2.9-4.8%) [15]. Because that confidence interval appears plausible, large inaccuracy is not evident from a fixed effects analysis of a process with large variation among clusters (I² approximately 98%) [14,15]. These examples highlight that one cannot rely on looking at a confidence interval for incidences of uncommon events to know if it is accurate. Rather, you need to understand how the confidence interval was calculated and rely on earlier simulation studies that tested the coverage of such confidence intervals.

Generalized estimating equations are another statistical method for estimating the marginal mean, but they are unsuitable for these data. They are robust to the mechanism of association within clusters (i.e., the binomial assumption). Li and Redden’s simulations showed that with 30 clusters, a mean of 50 patients per cluster, an underlying incidence proportion of 1.0%, and Student’s t-distribution used to calculate the confidence interval with 28 degrees of freedom (not 29, but the more suitable number of clusters minus two), the type I error rate was 5.4% (standard error 0.4%), no different from the nominal type I error rate of 0.05 [16]. The results of the generalized estimating equations for the regional anesthesia data were 1.67% (95% confidence interval, 1.54-1.80%) with ultrasound and 0.92% (0.83-1.03%) without ultrasound (Stata J). The point estimates are easy to follow, as these are the same as for the preceding jackknife approach. They are the ratio of the sum of events among all clusters in a group divided by the total sample size of the group. However, the confidence intervals are too narrow (e.g., 1.54-1.80%, unlike the one obtained using random-effects logistic regression: 1.06-3.27%). The reason is that the generalized estimating approach does not model heterogeneity among studies, which is unsuitable for the articles with I² values of 92% and 95%. Reiterating, when an analyst is presented with a meta-analysis, the unsuitability may be obvious, but often much less so when results for anesthesia data are presented in a different context, such as a management report [17].

Deviation of logits and probits from normal distributions: extra simulations

Our second dataset, included in the supplemental PDF file, is departmental clinical supervision scores (Stata K) [3]. Anesthesiology residents completed daily evaluations of the faculty anesthesiologists using the nine-item clinical supervision scale [3,18]. (The evaluations included obstetrical anesthesiology.) All nine items were completed for submission [19]. For consistency with the rest of our review and without loss of generality, we examine the proportions of scores with supervision quality below the desired level (i.e., small percentages) [3]. We previously demonstrated that random-effects logistic regression can be used to assess the overall quality of clinical supervision of trainees in the anesthesia department over a one-year period [3]. We also previously repeated the statistical studies using data from the University of Florida, Gainesville, FL, for purposes of evaluating (and showing) replicability [20]. The distributions of mean scores among raters had marked negative skewness and were inconsistent with normal distributions [3]. In contrast, analyses are performed using the logits of the proportions of evaluations with all nine items receiving the maximum possible score, calculated for each rater. The logits followed distributions sufficiently close to normal that we could use them in the random-effects modeling (Stata L) [3]. The probit-transformed data had a significantly poorer fit quality to a normal distribution. Parameters and confidence intervals were estimated using intercept-only random-effects logistic regression, and then inverses were computed to convert results from the logit scale to proportions (Stata M) [3].

We have used the conditional expectation (i.e., the median rater) to interpret departmental supervision performance [3]. The percentage of evaluations with a suboptimal score was 4.5% (95% confidence interval, 2.6-7.6%) in the later year, significantly less than the 9.9% (6.1-15.6%) during the earlier year, as shown in Reference [3]. Using paired analyses by ratee anesthesiologists, we also demonstrated that the odds of supervision quality being less than desired decreased with evaluation, notification of scores, and explanation of how to improve performance [21,22]. We performed an unpaired analysis using mixed-effects logistic regression, obtaining the same conclusion: an odds ratio of 0.44 (0.21-0.92, P = 0.028) (Stata N).

We consider three questions. First, would even a small deviation in the logits from a normal distribution be relevant? Second, would moderate deviation of the probits from a normal distribution be relevant? Third, can we trust the results of the generalized estimating equations? As context, the heterogeneity statistic I² equals approximately 95% (Stata O).

Supplemental Stata P has computer simulations. We set the “true” incidence proportion at 5.0%. We used the estimated variance in the logit scale from the N = 60 raters as the true value. We used the observed sample sizes for the 60 clusters. The harmonic mean was 34 per cluster, like for the meta-analysis of regional anesthesia procedures above. The coefficient of variation of the sample sizes among clusters was 65% [3], which is close to the 62% coefficient of variation for regional anesthesia procedures [2], as mentioned above. The correct coverage for the simulated 95% confidence intervals was 95.0%. The random-effects logistic regression performed nominally, with a 94.7% accuracy (standard error, 0.03%). The probit regression, which falsely assumed normality, did not achieve the expected performance: 92.5% (standard error, 0.04%) conditional and 91.6% (standard error, 0.04%) marginal. The generalized estimating equation approach calculated confidence intervals that included the true value for only 24.1% (standard error, 0.3%) of simulations. The method is unbiased but not reliable.

Applying the simulation results, we then calculated the marginal means from the random-effects logistic regression (Stata Q): 19.3% (95% confidence interval, 13.5-25.1%) for the earlier year and 11.8% (7.6-16.0%) for the later year. The confidence intervals were suitably wider than those obtained using the generalized estimating equations with these evaluation data (Stata R) [3]: 18.2% (17.2-19.2%) and 10.0% (9.2-10.8%).

Limitations

We have not reviewed an entirely different approach that can be used for incidence proportions and other anesthesia endpoints, but only when there are replications over time, marginal estimates are of interest, and quantifying the variation within and among clusters is unnecessary. Calculate the mean incidence for each period. The mean incidences are the summary measures. Next, calculate the mean and the standard error of the means among the summary measures for each period. For example, averaging over four-week or eight-week periods, the mean proportional incidences of case cancellations [23,24] are statistically independent of one another, just as are the total hours of cases [25,26], the numbers of cases [26,27], and the turnover times [28,29]. This batch means method is commonplace in industrial engineering, simulation output analysis, and engineering statistics. For example, using N = 28 four-week periods, 11.8% (standard error, 0.2%) of cases for which the patient was an inpatient preoperatively were cancelled after 7:00 am on the day before surgery. This approach depends on the sample size for each period being sufficiently large to have several events per period, the periods being of sufficient duration to be uncorrelated, the number of periods being sufficiently large to quantify variation among periods, and yet the periods being of sufficiently brief total time that the incidence proportion is stationary [23,28].

We have reviewed only the estimation of incidence proportions [30]. Austin simulated mixed-effects logistic regression models with non-normally distributed random effects [30]. His focus was analogous to our above comparison of studies with and without ultrasound guidance [30]. To understand his results, consider that whether ultrasound is used or not is a fixed effect term, while the studies are the random effect. There was no significant effect of deviations of the random effect from a normal distribution on the fixed effect’s coefficients’ type I error rates [30]. Thus, if the incidence of neurological dysfunction is the same between studies without and with ultrasound guidance, violations of the assumption that the logits follow a normal distribution matter little. In contrast, for a fixed effect coefficient that differed from zero, there was less than nominal coverage when there were few clusters (e.g., 25) and a small number of patients per cluster (e.g., 25) [30]. Austin simulated 50 and 25 clusters each with 50 and 25 patients per cluster [30]. For both, coverage was less than nominal (P < 0.0001) [30]. These results are consistent with the other reviewed articles and our simulations. The incidence proportion coverages we reviewed are unlike type I error rates because that would be analogous to zero in the logit scale, corresponding to incidence proportions of 50%. Rather, the incidence proportions we reviewed are comparable to fixed effects terms that truly differ substantively from zero on the logit scale. For these, the deviation of the random effect from a normal distribution can substantially affect results [30].

## Conclusions

Our conclusions align precisely with the goals listed in the Methods section. First, when estimating conditional and especially marginal estimates of incidence proportions, the probability distribution of the random effect matters for accurate confidence interval coverage when the events modeled are uncommon (e.g., adverse anesthesia events) and reported incidences are heterogeneous among clusters (e.g., for the distributions of anesthesia endpoints among studies, anesthesiologists, or hospitals). Intercept-only random-effects logistic regression is a good starting point for such analyses because these problems functionally are always complete data. We recommend using this method. Clusters (e.g., studies) with zero events in the numerator are addressed without the need for continuity corrections, even when there are many such clusters. Second, for some problems, marginal probability estimates are desired (e.g., incidence proportions of regional anesthesia complications). For other problems, conditional estimates are readily interpretable (e.g., perspectives of the median anesthesiology resident). The two can differ markedly for small proportions (e.g., ≤5%) when heterogeneity exists among clusters. We recommend knowing which one is being reported and, for each application, consider which would be preferred. Techniques that do not account for random effects (e.g., generalized estimating equations) can lead to biased estimates and inadequate confidence interval coverage. Therefore, we recommend using random effects and not falsely claiming that multiple observed zero events mean homogeneity. We hope that our examples have made clear that these principles for quantifying incidences and our recommendations apply not only to scientific studies (e.g., meta-analysis) but to routine hospital and anesthesia department quality reports.
